# Characterization of Peptide Utilization by *Bifidobacterium bifidum*: Insights From Computer Simulations and In Vitro Verification to Enhance Nitrogen Source Utilization

**DOI:** 10.1002/fsn3.71334

**Published:** 2025-12-11

**Authors:** Xin Qian, Zheming Gu, Xin Tang, Bingyong Mao, Qiuxiang Zhang, Jianxin Zhao, Wei Chen, Shumao Cui

**Affiliations:** ^1^ State Key Laboratory of Food Science and Resources Jiangnan University Wuxi China; ^2^ School of Food Science and Technology Jiangnan University Wuxi China; ^3^ International Joint Research Laboratory for Maternal‐Infant Microbiota and Health Jiangnan University Wuxi China; ^4^ National Engineering Research Center for Functional Food Jiangnan University Wuxi China

**Keywords:** *Bifidobacterium bifidum*, chemical bond, molecular docking, peptide utilization

## Abstract

*Bifidobacterium bifidum* ( *B. bifidum* ) exhibits limited efficiency in utilizing nitrogen sources, posing challenges for its industrial production. Preliminary investigations indicate that *B. bifidum* preferentially utilizes peptides over amino acids and proteins during its growth. Understanding the specific peptide utilization characteristics of *B. bifidum* is essential for addressing these production challenges. In this study, we hydrolyzed casein using various enzymes to prepare peptides, which were then used to culture *B. bifidum* strains. We identified peptides that are preferentially utilized by *B. bifidum* and analyzed their characteristics. The results demonstrated that *B. bifidum* favors peptides with molecular weights below 2000 Da, particularly those rich in proline, glutamic acid/glutamine, and leucine. Subsequently, we employed CDOCKER to dock the key peptide transport protein OppA with 2098 peptides. The docking results revealed that OppA binds peptides ranging from 2 to 15 residues in length, with molecular weights less than 2000 Da and containing proline or leucine, which is consistent with in vitro experimental results. Further analysis indicated that the length of transportable peptides correlates with the size of the OppA‐binding cavity. The presence of proline and leucine enhances interactions with OppA, thereby increasing affinity and preference for these amino acids in peptides. This study elucidates the peptide utilization characteristics of *B. bifidum* and explores the relationship between the OppA protein and peptide transport properties, providing theoretical insights for improving the growth and industrial production of *B. bifidum* .

## Graphical abstract

*Bifidobacterium bifidum* preferentially absorbs short peptides with molecular weights below 2000 Da that are rich in proline (Pro) and leucine (Leu). Molecular docking reveals that the length of transportable peptides correlates with the size of the OppA‐binding cavity, while the presence of Pro and Leu significantly enhances peptide‐OppA interactions, thereby increasing affinity and preference for these amino acids in peptides.

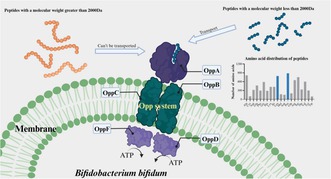

## 1 Introduction

As a probiotic, *B. bifidum* provides multiple health benefits, including alleviating colitis, alleviating Alzheimer's disease, and relieving constipation, making it a promising candidate for wide‐ranging applications in functional foods and dietary supplements (Khailova et al. 2009; Makizaki et al. 2021; Shamsipour et al. 2021). However, its industrial production is hindered by its limited efficiency in utilizing nitrogen sources. Studies have shown that *B. bifidum* has low cell wall protease activity, impeding effective protein utilization (Bottari et al. 2017). Additionally, due to a lack of various amino acid transporters, its amino acid utilization rate is inferior to that of peptides (Ferrario et al. 2015; Zafar and Saier Jr. 2022).

**FIGURE 1:**
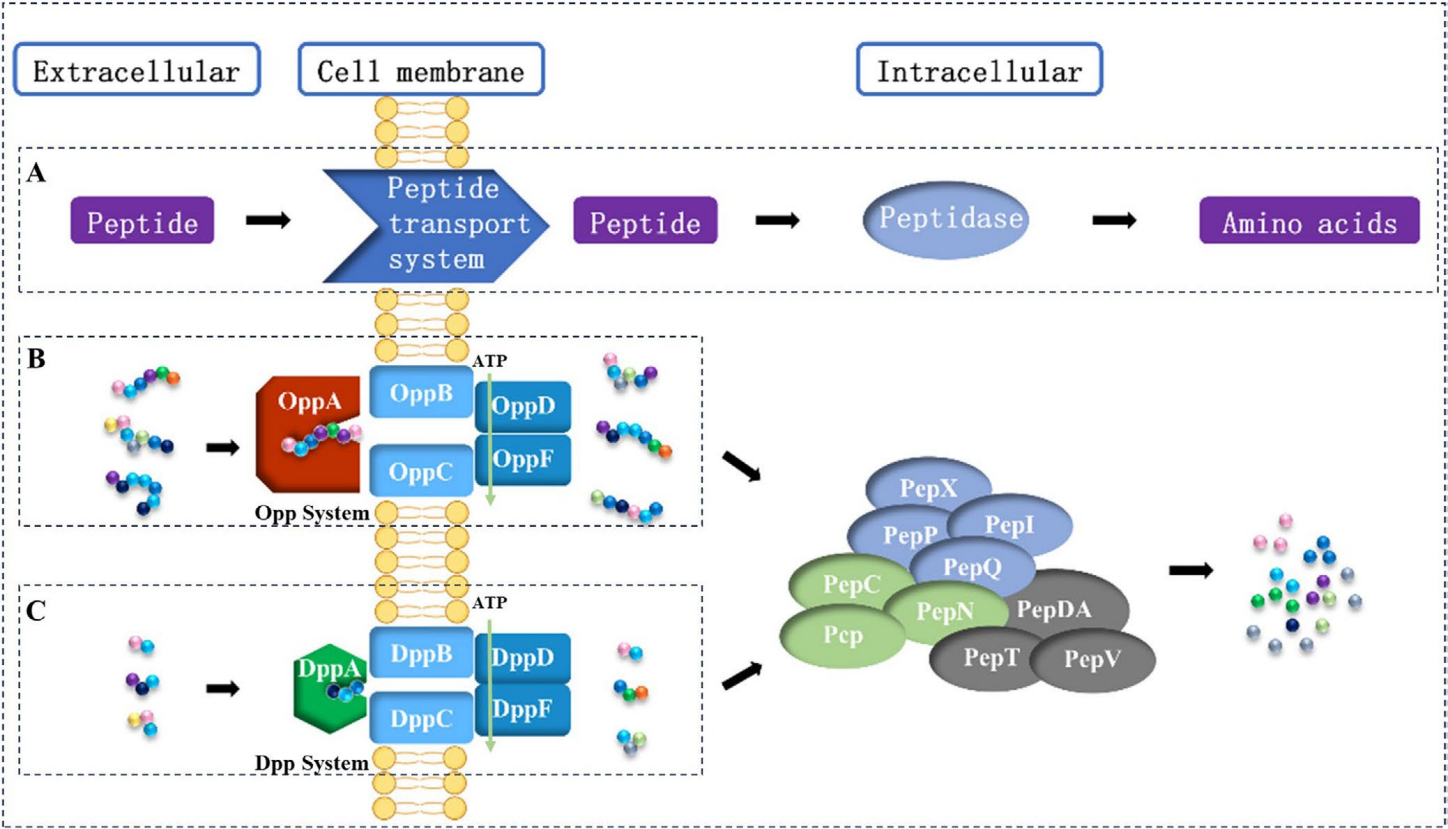
Peptide utilization mechanism of *Bifidobacterium*. (A) Schematic diagram of the general mechanism of peptide utilization in *Bifidobacterium*; (B) The peptide transporter Opp system of *Bifidobacterium*; (C) The peptide transporter Dpp system of *Bifidobacterium*.

The peptide transport system is crucial for *Bifidobacteria* to utilize environmental peptides (Figure 1). The peptide transport system in *Bifidobacteria* is primarily classified into two categories: (a) the Opp system of the ABC transporter superfamily, comprising oligopeptide‐binding protein OppA, osmotic enzymes OppB and OppC, and ATP‐binding proteins OppD and OppF (Norcross et al. 2019); and (b) the Dpp system of the ABC transporter superfamily, consisting of DppABCDF, which primarily transports dipeptides and tripeptides composed of hydrophobic branched‐chain amino acids (Foucaud et al. 1995). However, *B. bifidum* currently characterized strains exclusively possess the Opp system (Cui et al. 2022). However, not all species primarily rely on the Opp system to transport oligopeptides. Research has shown that a new peptide transport system OTS has been discovered in *Streptococcus thermophilus* (Nawara et al. 2016), while *Lactococcus lactis* has three peptide transport systems: DtpT, Opp, and DtpP. DtpP preferentially transports dipeptides and tripeptides composed of hydrophobic amino acids (Foucaud et al. 1995). *Helicobacter pylori* has Dpp and Opp systems and may have additional peptide transporters. In addition, in *Saccharomyces cerevisiae* , there are two different peptide transport mechanisms: one is the PTR system for dipeptides/tripeptides, and the other is the OPT system for tetrapeptides/pentapeptides (Melinda et al. 2001). These studies indicate that there is diversity in the peptide transport systems of different bacteria, reflecting their adaptability to different environments and nutritional requirements.

Reports indicate that peptide utilization by OppA follows a “Venus flytrap” mechanism, where OppA determines the specificity of peptide utilization within the Opp system (Lanfermeijer et al. 1999). Although different bifidobacteria exhibit varying peptide specificities, multiple studies have found a common preference for short peptides (Bottari et al. 2017; Cui et al. 2022). This variability is likely due to differences in OppA proteins among bifidobacteria. To elucidate the specific mechanism of peptide utilization by *B. longum* , molecular docking simulations revealed that *B. longum* KACC91563 prefers oligopeptides rich in proline and containing at least one leucine, isoleucine, or valine (Chai et al. 2022). However, there are limited reports on the peptide utilization characteristics and mechanisms in bifidobacteria. While some studies have provided insights into the peptide preferences of *B. bifidum* , the underlying reasons remain unexplained.

This study aims to summarize the specific characteristics of peptides preferred by *B. bifidum* from screened nitrogen sources, analyze the reasons for these preferences using bioinformatics methods. Our goal is to contribute to the industrial production of *B. bifidum* by providing a theoretical basis for the targeted preparation of efficient nitrogen sources based on bacterial characteristics.

## 2 Materials and Methods

### 2.1 Strain, Culture Medium, and Culture Conditions

*B. bifidum* strains CCFM16, FBJ1M4, FJSNT162, and FGZ612M3 were procured from the Culture Collection of Food Microbiology (CCFM) at Jiangnan University (Wuxi, China). The strains were inoculated in either nitrogen source screening medium or basic chemically defined medium (bCDM) (Petry et al. 2000) at pH 6.5, with a 2% (v/v) inoculation rate, and incubated anaerobically at 37°C until the end of the logarithmic phase.

The nitrogen source screening medium contained (per liter of distilled water): 1 g of nitrogen source, 5 g of glucose, 7 g of dipotassium phosphate, 7 g of disodium phosphate, and 1 g of cysteine hydrochloride, adjusted to pH 6.5.

### 2.2 Screening for Preferentially Utilized Nitrogen Sources

Four *B. bifidum* strains were cultured with 1 g/L of various nitrogen sources, including microbial (yeast extract FM803 and LP0021B), plant (soybean peptone FP410 and 763), animal (fish meal peptone and beef peptone), and milk‐based (casein peptone and tryptone). The nitrogen source screening medium was used as the control group, with 0.2 g/L yeast extract LP0021B, 0.4 g/L tryptone, and 0.4 g/L beef extract, based on the proportions found in MRS medium. Optical density at 600 nm (OD_600_) was measured at the end of the logarithmic phase.

### 2.3 Preparation of Casein Peptide

A casein solution was prepared at a concentration of 70 g/L, to which protease was added to achieve an enzyme activity of 2.8 × 10^5^ U/L. Enzymatic hydrolysis was performed at a constant pH in a parallel bioreactor. The enzymatic hydrolysis conditions, including temperature, enzyme types, and pH, are detailed in Table 1. Post‐hydrolysis, the solution was boiled for 10 min to inactivate the enzyme, cooled to room temperature, and adjusted to the isoelectric point (pI) of casein (4.6–4.8). The suspension was then centrifuged (8000×*g*, 20 min), and the supernatant was lyophilized to obtain casein peptides.

**TABLE 1:** Enzymatic hydrolysis conditions for different casein peptides.

Protease	Trypsin	Neutral protease	Papain	Flavor protease	Alkaline protease
Enzyme activity (U/g)	2.5 × 10^5^	5 × 10^5^	8 × 10^5^	3 × 10^4^	2 × 10^5^
Enzymatic hydrolysis temperature (°C)	50	50	50	50	50
Enzymatic hydrolysis pH	7.5	7	7	6.5	9

For ultrafiltration separation, an appropriate amount of peptide solution was added to a 50 mL ultrafiltration tube with a molecular weight cut‐off (MWCO) of 2000 Da. The solution was centrifuged multiple times (4000×*g*, 20 min, 4°C), and the filtrate was freeze‐dried.

We chose five enzymes (Alkaline protease, Flavor protease, Neutral protease, Papain, Trypsin) to hydrolyze casein, and the peptide solution was collected at five reaction time points (0.5, 1, 2, 4, 5 h), and a total of 30 different peptides were obtained. The peptides were classified into five categories according to the enzymes used to hydrolyze the proteins, including alkaline protease hydrolyzed casein product (CHA), flavor protease hydrolyzed casein product (CHF), neutral protease hydrolyzed casein product (CHN), papain hydrolyzed casein product (CHP), and trypsin hydrolyzed casein product (CHT). We have added numbers to these abbreviations to distinguish the products obtained at different digestion times. For example, CHA0.5 is the peptide obtained after 0.5 h of casein hydrolysis by alkaline protease.

### 2.4 Determination of the Growth‐Promoting Effect of Casein Peptide

Four *B. bifidum* strains were cultured in bCDM medium containing 4 g/L casein hydrolysate and 1 g/L cysteine until the end of the logarithmic phase. OD_600_ was measured to assess growth.

### 2.5 Determination of Peptide Molecular Weight

The molecular weight of peptides was determined using gel exclusion chromatography, following a modified method by Huang, Huo, et al. (2021). The analysis was performed on a TSKgel 2000 SWXL column (300 × 7.8 mm) with a mobile phase consisting of acetonitrile‐water‐trifluoroacetic acid (45:55:0.1) at a flow rate of 0.5 mL/min. UV detection was conducted at a wavelength of 220 nm.

### 2.6 Peptide Transport

The peptide transport assay followed a modified protocol by Charbonnel et al. (2003). Bacterial cultures at the end of the logarithmic phase were centrifuged (8000×*g*, 10 min, 4°C), washed three times with buffer A (0.1 mol/L K_3_PO_4_, 0.05 mol/L MgSO_4_, pH 6.5), and resuspended to A_650_ ≈ 25. An equal volume of 20 mmol/L 2‐deoxyglucose was added and incubated at 37°C for 30 min to deplete intracellular amino acids and peptides. The bacterial sludge was washed three times with buffer A and resuspended to A_650_ ≈ 50. Buffer B (buffer A with 5 g/L glucose) was added to achieve A_650_ ≈ 15, followed by the addition of 2.0 mg/mL peptide and incubation for 6 h. Samples were taken before and after incubation, centrifuged (8000×*g*, 10 min, 4°C), and the supernatant was freeze‐dried.

### 2.7 Determination of Amino Acid Consumption in Peptide Transport Experiment

Tryptophan was determined using the method described by Lv (2022) with slight modifications. Initially, the peptide was hydrolyzed using an alkaline solution, and its content was subsequently quantified via spectrophotometry. Specifically, 100.0 mg of peptide was placed in a 25 mL volumetric flask, followed by the addition of a 5% KOH solution to react for 18 h in an incubator at 40°C. The hydrolysate was then centrifuged (6000×*g*, 3 min, 4°C), and the supernatant was collected. Subsequently, 2 mL of the supernatant and 5 mL of a 1% *p*‐dimethylaminobenzaldehyde solution were added to a colorimetric tube, mixed thoroughly, and allowed to react at room temperature for 30 min. For blank samples, 2 mL of the supernatant and 5 mL of an 18.4 mol/L sulfuric acid solution were used. Standard reaction solutions consisted of 2 mL tryptophan solutions at various concentrations and 5 mL of an 18.4 mol/L sulfuric acid solution. After the reaction, 0.2 mL of a 0.2% sodium nitrite solution was added to each tube and mixed well. The absorbance at 590 nm was measured to determine the tryptophan content in the sample based on a standard curve.

The remaining 19 amino acids were quantified using a modified version of the method reported by Huang, He, et al. (2021). The peptide underwent acid hydrolysis by adding 100.0 mg of peptide and 6 mL of a 6 mol/L hydrochloric acid solution to hydrolysis tubes, which were then sealed and placed in an oven at 120°C for 22–24 h. The hydrolysate was evaporated to dryness and re‐dissolved in ultra‐pure water. For derivatization, 0.5 mL of the sample solution was mixed with 0.5 mL of a 14% triethylamine‐acetonitrile solution and 0.5 mL of a 1.2% phenyl isothiocyanate‐acetonitrile solution in a centrifuge tube, left at room temperature for 30 min. Subsequently, 1 mL of *n*‐hexane was added, vortexed, and centrifuged (8000×*g*, 5 min). The supernatant was filtered through a 0.22 μm membrane for HPLC analysis. The chromatographic conditions included a BEH C18 column (250 × 4.6 mm, 5 μm) with mobile phases consisting of a sodium acetate‐acetonitrile solution (97:3) and an acetonitrile‐water solution (80:20). The flow rate was set at 1.0 mL/min with gradient elution, and detection occurred at a wavelength of 254 nm.

### 2.8 Acquisition and Modeling of OppA

The peptide transport system (OppABCDF and DppABCDF) for *B. bifidum* was downloaded from the UniProt database (https://www.uniprot.org/). A local database was created using BLAST (v2.11.0) tools makeblastdb.exe and blastp.exe to mine genes.

The OppA sequence was uploaded to the WeMol website (https://wemol.wecomput.com/) for modeling using AlphaFold (v2.3.0) (Jumper et al. 2021), selecting the optimal model. The confidence level of the model was evaluated using the SAVES server (https://saves.mbi.ucla.edu/) with ERRAT (Colovos and Yeates 1993) and Verify 3D (Bowie et al. 1991; Luthy et al. 1992).

The structure of OppA expressed in *gene1021* of *B. bifidum* CCFM16 has been uploaded to the Missense 3D website (Ittisoponpisan et al. 2019) (http://missense3d.bc.ic.ac.uk/missense3d/) and mutations were defined using PyMOL (v2.6.0) to analyze their impact on protein structure.

### 2.9 Determination of the OppA Expression Levels of *B. bifidum*

*B. bifidum* was cultivated in MRS for 12 h. The fermentation broth was harvested by centrifugation (8000×*g*, 10 min) to collect cell pellets. Total RNA was isolated using the Vazyme R403 kit (OD_260_/OD_280_ ratio of 1.8–2.1). cDNA was synthesized with the Vazyme R433 reverse‐transcription kit and diluted 60‐fold for downstream analysis. Real‐time quantitative PCR (RT‐qPCR) was performed using the Vazyme Q711 kit. Primers were listed in Table S3; among them, 16S rRNA primers EUB338 and EUB518 were designed as described by Fierer et al. (2005). Following the method of Livak and Schmittgen (2001), the relative expression level of *OppA* was calculated, with *B. bifidum* CCFM16 used as the calibrator strain.

### 2.10 Molecular Docking

Data sets were downloaded from the ProBiS‐Fold peptide database (http://probis‐fold.insilab.org/datasets). Peptides containing only the 20 common amino acids were selected for molecular docking.

The peptides were assigned a CHARMM36 force field using Discovery Studio 2019 (v19.1.10), prepared using Prepare Ligands with pH parameters set to 6.0–7.0.

In Discovery Studio 2019, receptor proteins were prepared using Prepare Protein, selecting the largest cavity as the binding region. CDOCKER was employed for molecular docking predictions, setting Top Hits to 1 and Pose Cluster Radius to 0.5.

In this study, successful docking was considered to have occurred if, upon molecular docking of the ligand and receptor, a protein–peptide complex was formed and the ‐CDOCKER_ENERGY was > 0. In addition, this study defined the docking binding success rate as the percentage of successful docking between batches of peptides and OppA, which resulted in energetically favorable binding complexes. Docking binding success rate (%) = (Number of successful dockings/Total number of docking attempts) × 100%.

### 2.11 Data Statistics and Analysis

All experiments were repeated at least three times. Data plotting was performed using GraphPad Prism (version 9.0). Statistical significance was analyzed using one‐way ANOVA (Tukey test), with *p* < 0.05 indicating statistical significance.

## 3 Results

### 3.1 Screening of Preferentially Utilized Nitrogen Sources by *B. bifidum*

Four strains belonging to the *B. bifidum* species were cultured in various nitrogen sources to identify their preferentially utilized nitrogen sources (Table 2). The results indicated that milk‐based nitrogen sources were the most effective, although the utilization varied among the strains. Specifically, the OD_600_ values for strains CCFM16 and FBJ1M4 in tryptone were 0.347 and 0.383, respectively, representing increases of 9.8% and 2.4% compared to casein peptone. Conversely, strains FJSNT162 and FGZ612M3 exhibited better growth in casein peptone, with OD_600_ values of 0.446 and 0.342, respectively, which were 2.1% and 12.9% higher than in tryptone. Overall, *B. bifidum* exhibited the highest growth efficiency with milk‐based nitrogen sources, followed by animal‐derived, plant‐derived, and finally microbial‐derived nitrogen sources. Both casein peptones and tryptone are milk‐based nitrogen sources; casein peptones are acid/enzymatic hydrolysates of casein, while tryptone is tryptic digests of casein. This preference may be attributed to the prioritized utilization of peptides by *B. bifidum* , coinciding with the higher peptide abundance in milk‐derived nitrogen sources compared to other categories (Wang et al. 2021). Consequently, casein, a milk‐based nitrogen source, was selected for further investigation of nitrogen utilization characteristics in *B. bifidum* .

**TABLE 2:** Effects of different nitrogen sources on *Bifidobacterium bifidum* growth.

Origin	Name	CCFM16	FBJ1M4	FJSNT162	FGZ612M3
Microbial	Yeast extract LP0021B	0.087 ± 0.002^f^	0.095 ± 0.004^f^	0.113 ± 0.002^f^	0.089 ± 0.003^e^
	Yeast extract FM803	0.092 ± 0.005^f^	0.078 ± 0.007^g^	0.102 ± 0.004^f^	0.093 ± 0.009^e^
Plant	Soy peptone FP410	0.123 ± 0.005^e^	0.157 ± 0.007^d^	0.186 ± 0.008^e^	0.147 ± 0.005^d^
	Soy peptone 763	0.142 ± 0.007^e^	0.113 ± 0.003^e^	0.234 ± 0.010^d^	0.132 ± 0.005^d^
Animal	Fish meal peptone	0.276 ± 0.011^c^	0.268 ± 0.003^c^	0.403 ± 0.008^b^	0.287 ± 0.003^bc^
	Bovine bone peptone	0.213 ± 0.004^d^	0.303 ± 0.007^b^	0.324 ± 0.006^c^	0.268 ± 0.007^c^
Milk‐based	Casein peptone	0.316 ± 0.007^b^	0.374 ± 0.002^a^	0.446 ± 0.005^a^	0.342 ± 0.012^a^
	Tryptone	0.347 ± 0.011^a^	0.383 ± 0.008^a^	0.437 ± 0.009^a^	0.303 ± 0.006^b^

*Note:* Values in the table represent OD_600_ measurements. Results are presented as mean ± standard deviation. Superscript letters (a, b, c, d, e, f) above numbers indicate statistically significant differences between groups (*p* < 0.05).

### 3.2 Screening of Casein Hydrolysate to Promote the Growth of *B. bifidum*

**FIGURE 2:**
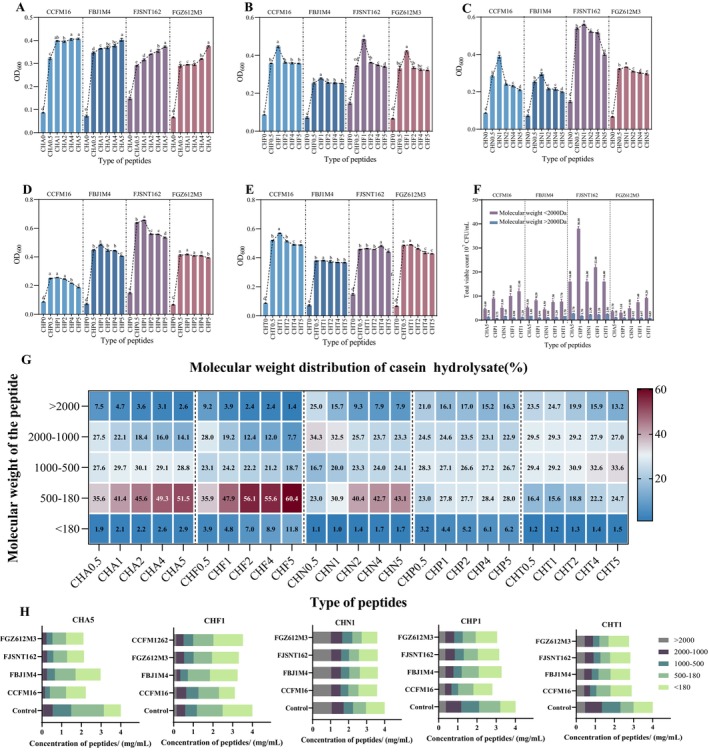
Growth of *Bifidobacterium bifidum* and molecular weight distribution of nitrogen source; (A) Effect of alkaline protease hydrolysis of casein hydrolysate on bacterial growth; (B) Effect of flavor protease hydrolysis of casein hydrolysate on bacterial growth; (C) Effect of neutral protease hydrolysis of casein hydrolysate on bacterial growth; (D) Effect of papain hydrolysis of casein hydrolysate on bacterial growth; (E) Effect of trypsin hydrolysis of casein hydrolysate on bacterial growth; (F) Effect of five optimal casein hydrolysates with different molecular weights on the biomass of *B. bifidum* ; (G) Molecular weight distribution of casein hydrolysate; (H) Concentration of peptides with different molecular weights before and after fermentation. The superscripts (a, b, c, d, e, f) above the columns in Figures 2A‐E indicate statistically significant differences (*p* < 0.05) between the groups.

We hydrolyzed casein using five different enzymes (alkaline protease, flavored protease, neutral protease, papain, and trypsin) at various hydrolysis times, resulting in 25 distinct casein hydrolysates. These hydrolysates were then used to culture four strains of *B. bifidum* , with casein serving as the control group (Figure 2A–E). The results demonstrated that casein hydrolysates enhanced the growth of the strains compared to casein alone. However, the growth‐promoting effects varied depending on the preparation process. For alkaline proteases, the longer the hydrolysis time, the more growth‐promoting the peptide obtained. The peptide CHA5 obtained from 5 h of hydrolysis had the best effect, and then as the hydrolysis time decreases, the effect decreased sequentially, and the peptide CHA0.5 obtained from 0.5 h of hydrolysis had the weakest promotional effect. For hydrolysates produced with the other four enzymes, a hydrolysis time of 1 h yielded the best growth promotion.

The choice of hydrolytic enzyme and hydrolysis duration significantly influenced the peptide and amino acid composition and molecular weight distribution of the casein hydrolysate. We measured these distributions for all 25 nitrogen sources (Figure 2G). The data revealed that longer hydrolysis times reduced peptide molecular weights and increased free amino acid concentrations. A correlation was observed between peptide molecular weight and bacterial growth for CHA hydrolysates. In contrast, flavor protease (CHF) produced many amino acids after extensive hydrolysis, reducing peptide content and growth promotion. For the other three enzymes (CHN, CHP, CHT), longer hydrolysis times decreased peptide molecular weights and growth levels, suggesting that prolonged hydrolysis diminishes the growth‐promoting effects by degrading preferred peptides.

These findings suggest that *B. bifidum* preferentially utilizes peptides of specific lengths and amino acid compositions. We selected five casein hydrolysates (CHA5, CHF1, CHN1, CHP1, CHT1) that effectively promoted *B. bifidum* growth for further analysis of peptide utilization characteristics.

### 3.3 Molecular Weight Distribution of Preferentially Utilized Peptides by *B. bifidum*

Four strains of *B. bifidum* were cultivated until the end of the logarithmic phase with bCDM medium containing 4 g/L peptide, 1 g/L cysteine for each strain. To identify which molecular weight peptides *B. bifidum* utilized, we measured changes in peptide content of different molecular weights before and after cultivation (Figure 2H). The results showed that all five types of casein hydrolysates were utilized, with the highest reduction observed in CHA5 peptides. Peptides larger than 2000 Da and amino acids smaller than 180 Da showed minimal decrease post‐fermentation. While utilization varied among strains, all preferred peptides were in the 180–2000 Da range.

Subsequently, we employed a 2000 Da ultrafiltration tube to segregate peptides into two fractions: those with molecular weights above 2000 Da and those between 0 and 2000 Da. These fractions were then used to culture *B. bifidum* . The results, depicted in Figure 2F, indicate that the peptides with molecular weights below 2000 Da exhibited superior utilization efficiency for *B. bifidum* strains CCFM16, FBJ1M4, FJSNT162, and FGZ612M3. Specifically, the biomass achieved was (1.2 ± 0.1) × 10^8^, (8.2 ± 0.5) × 10^7^, (3.8 ± 0.9) × 10^8^, and (9.2 ± 0.4) × 10^7^ CFU/mL, respectively, which were 9.92, 8.28, 22.09, and 8.23 times higher than their corresponding peptides above 2000 Da. This further corroborates that *B. bifidum* preferentially utilizes peptides with molecular weights of 2000 Da or less. Previous studies have shown that lactic acid bacteria YC‐380 (a 1:1 mixture of *S. thermophilus* and *Lactobacillus delbrueckii* subsp. *bulgaricus*) exhibit a similar growth trend with peptides below 3000 Da (Zhang et al. 2014). Although *L. delbrueckii* subsp. *bulgaricus* contains extracellular proteases (Shuang et al. 2015), which may explain its specific mechanism of peptide utilization in relation to *B. bifidum*, it is different, but both studies indicate that peptides below a specific molecular weight are more beneficial for bacterial growth, suggesting the universal advantage of low molecular weight peptides in promoting bacterial growth.

### 3.4 Amino Acid Composition of Preferentially Utilized Peptides by *B. bifidum*

The aforementioned results demonstrate that CHT1 and CHP1 peptides with molecular weights below 2000 Da optimally promote the growth of *B. bifidum* strains CCFM16, FBJ1M4, FJSNT162, and FGZ612M3, respectively. Consequently, these peptides were selected as the optimal nitrogen sources for further experimentation.

**FIGURE 3:**
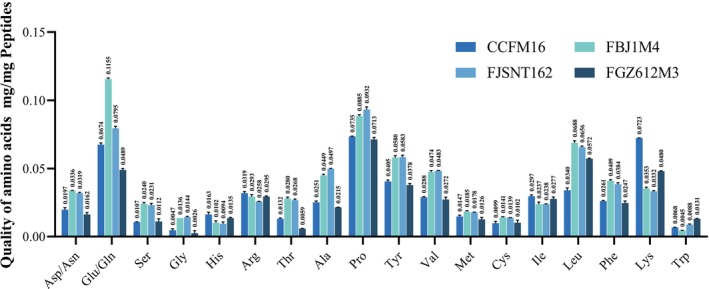
Composition of amino acids in preferentially utilized peptides by *Bifidobacterium bifidum* . The amino acid composition of peptides reduced in the culture medium before and after the peptide transport experiment of *B. bifidum* . The four colored columns represent CCFM16, FBJ1M4, FJSNT162, FGZ612M3.

We conducted peptide transport experiments using these optimal nitrogen sources and measured the amino acid composition before and after fermentation to elucidate the amino acid distribution in the preferentially utilized peptides of *B. bifidum* . The results indicate that FBJ1M4 primarily utilizes peptides containing glutamic acid/glutamine, followed by proline and leucine. CCFM16 and FGZ612M3 preferentially utilize peptides containing proline, glutamic acid/glutamine, lysine, and leucine. FJSNT162 favors peptides with proline, glutamic acid/glutamine, and leucine (Figure 3). Thus, in the four tested strains of *B. bifidum* (CCFM16, FBJ1M4, FJSNT162, FGZ612M3), proline, glutamic acid/glutamine, and leucine are prevalent in the preferentially utilized peptides of *B. bifidum* .

### 3.5 Prediction of Peptide Utilization Characteristics by Molecular Docking

**FIGURE 4:**
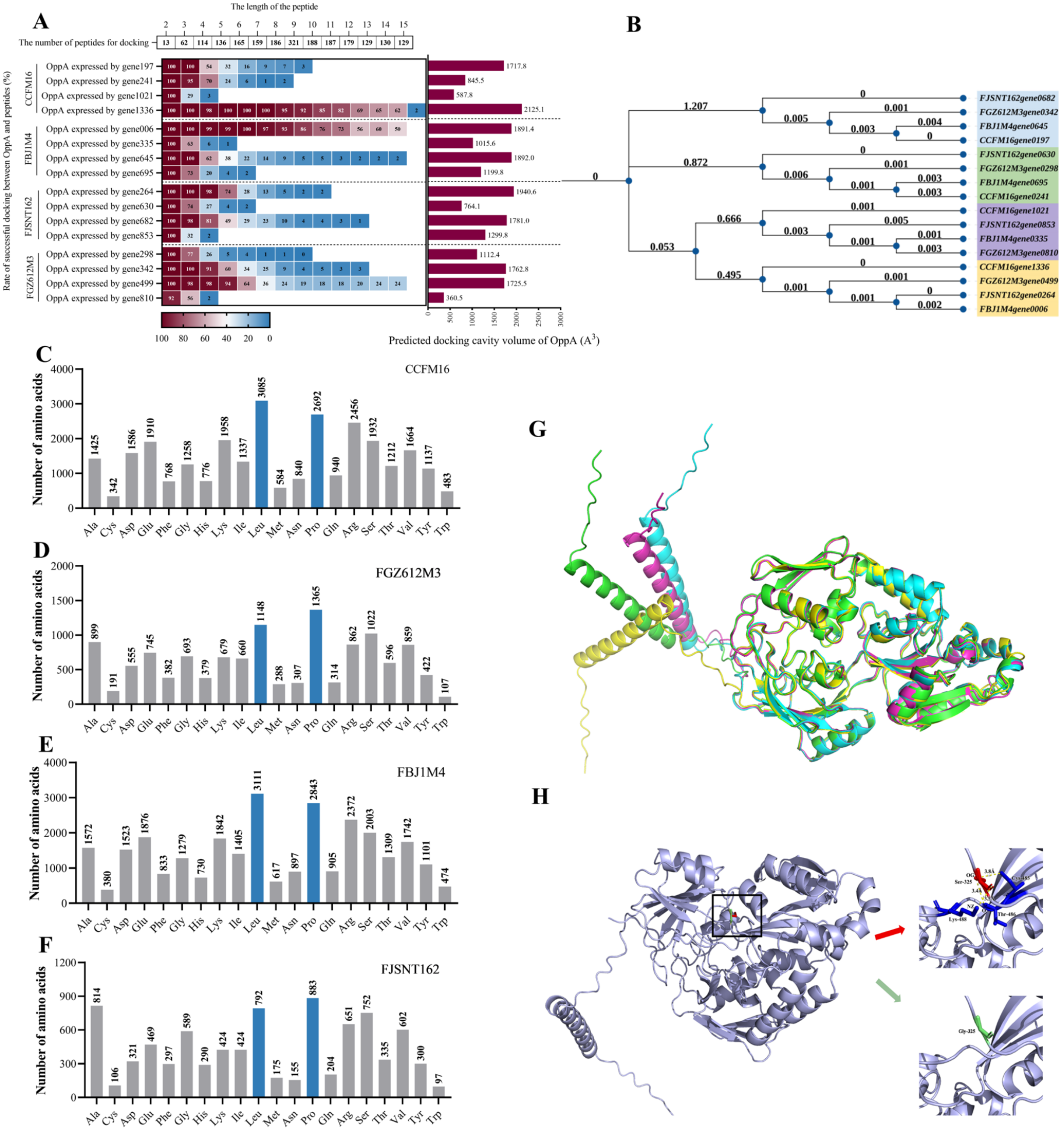
Results on predicting peptide utilization preferences of *Bifidobacterium bifidum* by simulating the binding of OppA and peptides using computer simulation; (A) Rate of successful docking between OppA and peptides and predicted docking cavity volume of OppA; (B) Neighbor‐Joining tree between genes expressing OppA proteins in four strains of *B. bifidum* (Disregarding the length of the branch tree, numbers represent genetic distance, and the smaller the genetic differences between numerical genes); (C) Composition of amino acids in peptides successfully docked with OppA of *B. bifidum* CCFM16; (D) Composition of amino acids in peptides successfully docked with OppA of *B. bifidum* FBJ1M4; (E) Composition of amino acids in peptides successfully docked with OppA of *B. bifidum* FGZ612M3; (F) Composition of amino acids in peptides successfully docked with OppA of *B. bifidum* FJSNT162; (G) Comparison of OppA protein structures corresponding to gene expression in four strains of *B. bifidum* . The OppA expressed by *CCFM16gene1021*, green; The OppA expressed by *FBJ1M4gene0335*, cyan; The OppA expressed by *FJSNT162gene0853*, purple; The OppA expressed by *FGZ612M3gene0810*, yellow; (H) Effect of amino acid 325 mutation on the OppA expressed by *CCFM16gene1021*.

It has been shown that OppA proteins play a crucial role in determining the specificity of bacterial peptide uptake. Although in vitro experiments can accurately characterize the binding properties of OppA (Klepsch et al. 2011; Tame et al. 1994), computer simulations provide a fast and convenient method of prediction (Chai et al. 2022; Ting and Rebecca 2002). Therefore, we obtained all amino acid reference sequences related to the OppA protein in *B. bifidum* from the UniProt database, translated the whole genome sequencing data of four experimental strains, and compared them with the reference sequence through the BLAST program to identify OppA homologous proteins in each strain. The comparison results are shown in Table S1, and Figure S1 also illustrates the operon diagram of the Opp system of *B. bifidum* using CCFM16 as an example. RT‐qPCR validation confirmed that the *OppA* genes of all strains listed in Table S1 are expressed during the logarithmic growth phase (Figure S2). Based on the positive sequence results obtained through comparison, AlphaFold was used for 3D structural modeling. Subsequently, we obtained 2098 peptide sequences from the ProBiS Fold Database, a repository compiling experimentally validated peptides reported in prior studies across multiple species. Using Discovery Studio 2019, we conducted CDOCKER molecular docking between each *B. bifidum* strain and these peptide segments (Figure 4A). The molecular docking predicted that OppA from each *B. bifidum* strain binds peptides 2–12 residues in length with a maximum of 15 peptides.

Significant differences were observed among different OppAs in terms of peptide docking. For instance, in CCFM16, the proteins expressed by *gene0197*, *gene241*, *gene1021*, and *gene1336* could bind peptides with maximum lengths of 9, 8, 3, and 15 residues, respectively. The highest molecular weight peptide successfully docked was 1860.2 Da (Table 3), which aligns with cultivation data from four tested strains of *B. bifidum* (CCFM16, FBJ1M4, FJSNT162, FGZ612M3) and further indicates that these four *B. bifidum* preferred peptides below 2000 Da.

**TABLE 3:** Molecular weight distribution of peptides preferred by *Bifidobacterium bifidum* .

Length	CCFM16 (Da)	FBJ1M4 (Da)	FJSNT162 (Da)	FGZ612M3 (Da)
2	170.2–294.3	170.2–294.3	170.2–294.3	170.2–294.3
3	241.3–469.5	241.3–469.5	241.3–469.5	241.3–469.5
4	270.3–595.7	270.3–595.7	270.3–595.7	270.3–595.7
5	327.3–736.8	327.3–736.8	327.3–685.8	327.3–736.8
6	426.5–907	426.5–907	426.5–852	426.5–785
7	497.5–1107.3	497.5–980.1	497.5–861.9	497.5–905
8	540.6–1088.3	540.6–1074.3	540.6–901.1	540.6–1029.1
9	639.7–1380.6	639.7–1255.4	639.7–1255.4	639.7–1145.2
10	682.7–1461.4	682.7–1353.5	682.7–1085.3	682.7–1230.4
11	781.9–1464.6	781.9–1464.6	781.9–1282.5	781.9–1342.3
12	1059.2–1651.7	1059.2–1582.7	1111.3–1344.4	1105.2–1536.8
13	1129.2–1774.1	1129.2–1724.1	—	1129.2–1601.7
14	995.1–1910.2	995.1–1860.2	—	995.1–1860.2
15	1462.7–1565.6	—	—	—

We also analyzed the amino acid composition of these successfully docked peptides (Figure 4C–F). The results show that *B. bifidum* CCFM16 and FBJ1M4 prefer peptides rich in leucine, followed by proline and arginine; FJSNT162 favors proline, alanine, and leucine; FGZ612M3 prefers proline, leucine, and serine. Overall, these strains exhibit a preference for peptides containing high levels of leucine or proline.

In summary, based on molecular docking results, the successfully docked peptides are composed of 2–15 residues with molecular weights less than 2000 Da and contain proline or leucine, aligning with our experimental findings.

### 3.6 Analysis of Relationship Between OppA and Molecular Weight of Preferentially Utilized Peptides by Molecular Docking

The whole genome analysis revealed the presence of the OppA system in four strains of *B. bifidum* , with each strain encoding four distinct OppA proteins (Table 4). The amino acid sequences of these OppA proteins were obtained and modeled using AlphaFold. Subsequently, the cavity volumes were calculated using Discovery Studio. The bar chart on the right side of Figure 4A illustrates the predicted internal cavity volumes of OppA proteins. A significant correlation was observed between the distribution of docking binding success rates from molecular docking simulations and the cavity volume size of OppA. Furthermore, a corresponding relationship appears to exist among OppA proteins from different strains. The OppA gene sequences were aligned using MEGA11 and the bootstrap method (10,000 times) to construct a neighbor‐joining (NJ) tree, which was visualized using iTOL (https://itol.embl.de/) (Figure 4B). The results clearly indicate that OppA proteins in *B. bifidum* are categorized into four types, with varying cavity volumes influencing the lengths of peptides they can transport. Notably, the OppA proteins expressed by *CCFM16gene1336*, *FBJ1M4gene0006*, *FJSNT162gene0264*, and *FGZ612M3gene0449* were modeled using AlphaFold (v2.3.0) and evaluated for confidence levels using ERRAT and Verify 3D (Table S2). The structural similarity among these OppA proteins was notably high, with only minor variations in the tail alpha helix angles (Figure 4G).

**TABLE 4:** Distribution of peptide transporters in different *Bifidobacterium bifidum* strains.

Gene name	Protein name	CCFM16	FBJ1M4	FJSNT162	FGZ612M3
*OppA*	Oligopeptide binding protein	4	4	4	4
*OppB*	Permease	3	3	3	3
*OppC*	Permease	2	2	2	2
*OppD*	ATP binding protein	2	3	3	3
*OppF*	ATP binding protein	3	2	2	2

Interestingly, despite the OppA expressed by *CCFM16gene1021* and *FBJ1M4gene0335* belonging to the same class and differing by only seven amino acids, their cavity volumes varied by 427.8 Å^3^. We hypothesize that amino acid mutations influence cavity volume, thereby affecting peptide transport. Using Missense 3D software, we simulated a mutation at the 325th amino acid Gly in the OppA expressed by *CCFM16gene1021* to Ser, as found in the OppA expressed by *FBJ1M4gene0335* (Figure 4H). This mutation aimed to investigate its impact on the peptide binding region area of the strains. The results indicated that the Ser_325_ parent structure in the OppA expressed by *CCFM16gene1021* was originally exposed with an RSA (Relative Solvent Accessibility, RSA) of 10% uncharged, forming three hydrogen bonds with other residues. Post‐mutation, Gly_325_ exhibited an RSA of 26.1% uncharged without hydrogen bonding interactions, expanding the protein cavity volume by 85.536 Å^3^. This suggests that amino acid site variations lead to changes in cavity volume, influencing peptide interactions and utilization among different strains of the same species.

In summary, the transportable peptide length is correlated with the size of the OppA binding cavity. Each *B. bifidum* strain possesses four distinct OppA proteins with varied peptide binding characteristics, collectively determining overall peptide utilization properties. Genetic factors causing amino acid changes in proteins likely contribute significantly to differences in peptide utilization among *B. bifidum* strains.

### 3.7 Analysis of Interaction Between OppA and Amino Acids by Molecular Docking

**FIGURE 5:**
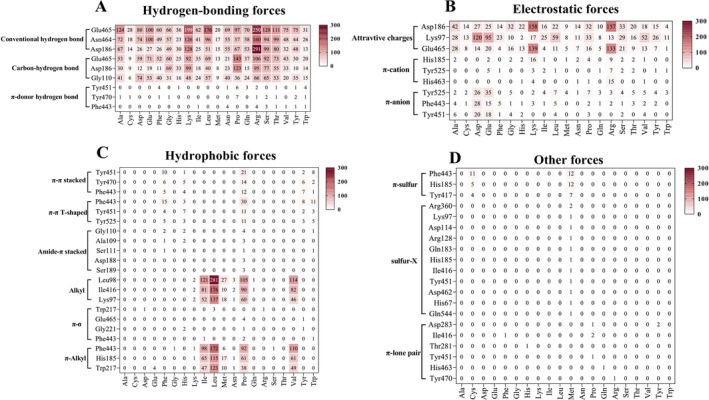
Interaction force between OppA and peptides; (A) Hydrogen‐bonding forces generated by docking peptides containing proline or leucine with OppA; (B) Electrostatic forces generated by docking peptides containing proline or leucine with OppA; (C) Hydrophobic forces generated by docking peptides containing proline or leucine with OppA; (D) Other forces generated by docking peptides containing proline or leucine with OppA.

A statistical analysis was conducted on interaction forces between peptides and OppA proteins during docking. Using the peptide‐binding protein OppA (expressed by CCFM16*gene1336*) as a representative example, we analyzed hydrogen bonding, electrostatic, hydrophobic, halogen, and other interactions (Figure 5). Conventional hydrogen bonding revealed that Arg on the peptide forms multiple connections with amino acids on the OppA protein, while proline forms more C‐hydrogen bonds with OppA; π‐donor hydrogen bonds were relatively minimal, with proline as the main acceptor (Figure 5A). Electrostatic forces included attractive charges, π‐anion forces, and π‐cation forces, primarily occurring between Lys, Arg, Glu, Asp with opposite charges (Figure 5B). Hydrophobic forces such as π–π stacking, alkyl force, and π‐alkyl force mainly occurred between leucine, proline, isoleucine, and valine on OppA proteins and peptides (Figure 5C). The five‐membered ring of proline and the carbon atoms of leucine were critical structural components mediating hydrophobic interactions. Other forces like π‐sulfur, sulfur‐X, and π‐lone pair interactions between the OppA expressed by *CCFM16gene1336* and amino acids in peptides were less frequent, primarily involving sulfur‐containing amino acids cysteine and methionine (Figure 5D).

Interaction forces between the OppA expressed by *CCFM16gene1336* and peptides are primarily hydrogen bonding, hydrophobic, and electrostatic forces, with proline, leucine, and arginine being the key amino acids involved. Peptides enriched with proline or leucine exhibit increased affinity for the OppA protein of *B. bifidum* due to the generation of more binding interactions. Consequently, such peptides are more efficiently transported by OppA and subsequently utilized.

## 4 Discussion

This study elucidates the characteristics of peptides preferentially utilized by *B. bifidum* and explores the relationship between the key protein OppA and these peptide utilization features. The growth challenges of *B. bifidum* , particularly in terms of nitrogen source utilization, have long been a concern in the industry. Previous research has identified issues such as cysteine deficiency (Ferrario et al. 2015), weak cell wall protease activity (Bottari et al. 2017), lack of amino acid transporters (Zafar and Saier Jr. 2022), and growth inhibition by hydrophobic amino acids (Gu et al. 2024). In order to gain a more comprehensive understanding of these challenges, future research should delve into the interactions between these factors, whether there are correlations or mutual influences. You can also focus on exploring how to address some of these challenges in a targeted manner.

Studies have shown that peptides promoting bacterial growth can be obtained through enzymatic hydrolysis and protein separation (Etoh et al. 2000; Li et al. 2021). Our research group previously employed chromatography to fractionate casein hydrolysate into components of varying sizes, ultimately identifying that components with molecular weights below 2000 Da promote better bacterial growth. Additionally, a preference for proline‐containing peptides was observed (Cui et al. 2022). This study confirms these findings, but using molecular docking technology to predict the binding of OppA to peptides provides a deeper explanation. Similar research on *B. longum* KACC91563 demonstrated a preference for oligopeptides rich in proline and containing leucine, isoleucine, or valine (Chai et al. 2022), aligning with our findings for *B. bifidum* . This suggests that different *Bifidobacterium* species may have similar peptide preference patterns, reflecting their adaptation to nutrients in similar intestinal microenvironments. However, it is worth noting that although OppA plays an important role in the metabolism of *Bifidobacteria* and even *lactic acid bacteria* (Frank et al. 2000), we can infer the nitrogen utilization characteristics of specific strains by studying the docking characteristics of OppA with peptides. But not all species are suitable for this prediction method of nitrogen utilization characteristics. In the plant pathogen Xanthomonas (Elisa et al. 2010), the OppA protein appears to have no significant effect on the uptake of oligopeptides, indicating that the function of OppA may vary by species, and not all OppA proteins are involved in peptide transport.

We also want to emphasize that in this study, computer simulation technology was mainly used as a predictive tool to identify peptides that preferentially bind to *B. bifidum* . Although molecular docking simulations provide valuable insights into the interaction between peptides and OppA, it is important to recognize the limitations of these simulations. Molecular docking is a commonly used computer‐aided drug discovery method (Luca and Giulio 2019; Shadi et al. 2023) that can be used to predict the binding mode between small molecules and target proteins (Fangfang et al. 2024). However, computer simulation relies on simplified models and parameters, which may not fully capture complex biological processes within the body. For example, simulations typically do not consider solvent effects, protein dynamics, or interactions with other cellular components, which may significantly affect peptide binding and transport. Additionally, the OppA follows the Venus flytrap mechanism, exhibiting both open and closed states (Yang et al. 2024). Molecular docking relies on rigid protein models from AlphaFold and does not account for this conformational change in OppA. Therefore, computer simulation results should be considered predictive and need to be validated experimentally. This limitation should be noted in future research.

This study advances our understanding of the peptide utilization characteristics in *B. bifidum* and addresses the nitrogen source utilization problem. However, further research is needed to develop targeted preparation methods for these peptides. Future studies should focus on the specific preparation processes for preferred peptides to develop efficient nitrogen sources, thereby significantly enhancing the nitrogen source utilization efficiency and biomass of *Bifidobacterium*.

## 5 Conclusion

This study identified a casein‐derived nitrogen source selectively utilized by *B. bifidum* . By preparing various casein hydrolysates for strain cultivation, we analyzed peptide utilization preferences and found that *B. bifidum* favors peptides with molecular weights below 2000 Da, rich in proline and leucine. Molecular docking of the key protein OppA with 2098 peptides revealed consistent peptide utilization characteristics with experimental results. The study also established a correlation between OppA cavity volume and peptide transport length, showing that proline and leucine in peptides enhance affinity with OppA. This research clarifies the peptide utilization characteristics of *B. bifidum* and contributes to solving its nitrogen source utilization problem.

## Author Contributions

**Xin Qian:** formal analysis, methodology, writing – review and editing. **Zheming Gu:** formal analysis, methodology, writing – original draft. **Xin Tang:** formal analysis, supervision. **Bingyong Mao:** methodology, project administration, writing – review and editing. **Qiuxiang Zhang:** supervision. **Jianxin Zhao:** funding acquisition. **Wei Chen:** funding acquisition. **Shumao Cui:** conceptualization, funding acquisition.

## Conflicts of Interest

The authors declare no conflicts of interest.

## Supporting information


**Data S1:** fsn371334‐sup‐0001‐DataS1.docx.

## Data Availability

The data that support the findings of this study are available from the corresponding author upon reasonable request.
