# Supplementary material for: Characterization of Peptide Utilization by Bifidobacterium bifidum: Insights From Computer Simulations and In Vitro Verification to Enhance Nitrogen Source Utilization
Source: Food Sci Nutr. 2025 Dec 11;13(12):e71334. doi: 10.1002/fsn3.71334 (PMC12698387; doi:10.1002/fsn3.71334)
Supplement: Supplementary file 1 — Data S1: fsn371334‐sup‐0001‐DataS1.docx. [file FSN3-13-e71334-s001.docx]

**Table S1** Genetic information of Opp system in *B. bifidum*

|  | Gene names | Gene | The function of proteins expressed by genes |
| --- | --- | --- | --- |
| CCFM16 | *gene0197* | *OppA* | OppA Oligopeptide-binding protein |
|  | *gene0198* | *OppD* | Oligopeptide transport ATP-binding protein |
|  | *gene0199* | *OppC* | Oligopeptide transport system permease protein |
|  | *gene0200* | *OppB* | Oligopeptide transport system permease protein |
|  | *gene0238* | *OppD* | Oligopeptide transport ATP-binding protein |
|  | *gene0239* | *OppF* | Oligopeptide transport ATP-binding protein |
|  | *gene0240* | *OppB* | Oligopeptide transport system permease protein |
|  | *gene0241* | *OppA* | OppA Oligopeptide-binding protein |
|  | *gene1021* | *OppA* | OppA Oligopeptide-binding protein |
|  | *gene1336* | *OppA* | OppA Oligopeptide-binding protein |
|  | *gene1337* | *OppB* | Oligopeptide transport system permease protein |
|  | *gene1338* | *OppC* | Oligopeptide transport system permease protein |
|  | *gene1339* | *OppF* | Oligopeptide transport ATP-binding protein |
|  | *gene1340* | *OppF* | Oligopeptide transport ATP-binding protein |
| FBJ1M4 | *gene0002* | *OppF* | Oligopeptide transport ATP-binding protein |
|  | *gene0003* | *OppF* | Oligopeptide transport ATP-binding protein |
|  | *gene0004* | *OppC* | Oligopeptide transport system permease protein |
|  | *gene0005* | *OppB* | Oligopeptide transport system permease protein |
|  | *gene0006* | *OppA* | OppA Oligopeptide-binding protein |
|  | *gene0335* | *OppA* | OppA Oligopeptide-binding protein |
|  | *gene0645* | *OppA* | OppA Oligopeptide-binding protein |
|  | *gene0647* | *OppD* | Oligopeptide transport ATP-binding protein |
|  | *gene0648* | *OppC* | Oligopeptide transport system permease protein |
|  | *gene0649* | *OppB* | Oligopeptide transport system permease protein |
|  | *gene0691* | *OppD* | Oligopeptide transport ATP-binding protein |
|  | *gene0692* | *OppD* | Oligopeptide transport ATP-binding protein |
|  | *gene0693* | *OppC* | Oligopeptide transport system permease protein |
|  | *gene0695* | *OppA* | OppA Oligopeptide-binding protein |
| FJSNT162 | *gene0260* | *OppF* | Oligopeptide transport ATP-binding protein |
|  | *gene0261* | *OppF* | Oligopeptide transport ATP-binding protein |
|  | *gene0262* | *OppC* | Oligopeptide transport system permease protein |
|  | *gene0263* | *OppB* | Oligopeptide transport system permease protein |
|  | *gene0264* | *OppA* | OppA Oligopeptide-binding protein |
|  | *gene0630* | *OppA* | OppA Oligopeptide-binding protein |
|  | *gene0632* | *OppB* | Oligopeptide transport system permease protein |
|  | *gene0633* | *OppD* | Oligopeptide transport ATP-binding protein |
|  | *gene0634* | *OppD* | Oligopeptide transport ATP-binding protein |
|  | *gene0678* | *OppB* | Oligopeptide transport system permease protein |
|  | *gene0679* | *OppC* | Oligopeptide transport system permease protein |
|  | *gene0680* | *OppD* | Oligopeptide transport ATP-binding protein |
|  | *gene0682* | *OppA* | OppA Oligopeptide-binding protein |
|  | *gene0853* | *OppA* | OppA Oligopeptide-binding protein |
| FGZ612M3 | *gene0298* | *OppA* | OppA Oligopeptide-binding protein |
|  | *gene0299* | *OppB* | Oligopeptide transport system permease protein |
|  | *gene0300* | *OppD* | Oligopeptide transport ATP-binding protein |
|  | *gene0301* | *OppD* | Oligopeptide transport ATP-binding protein |
|  | *gene0339* | *OppB* | Oligopeptide transport system permease protein |
|  | *gene0340* | *OppC* | Oligopeptide transport system permease protein |
|  | *gene0341* | *OppD* | Oligopeptide transport ATP-binding protein |
|  | *gene0342* | *OppA* | OppA Oligopeptide-binding protein |
|  | *gene0495* | *OppF* | Oligopeptide transport ATP-binding protein |
|  | *gene0496* | *OppF* | Oligopeptide transport ATP-binding protein |
|  | *gene0497* | *OppC* | Oligopeptide transport system permease protein |
|  | *gene0498* | *OppB* | Oligopeptide transport system permease protein |
|  | *gene0499* | *OppA* | OppA Oligopeptide-binding protein |
|  | *gene0810* | *OppA* | OppA Oligopeptide-binding protein |

**Table S2** Confidence evaluation of the peptide-binding protein model of *B. bifidum*

| Strain | Peptide binding protein | ERRAT | Verify 3D |
| --- | --- | --- | --- |
| CCFM16 | CCFM16gene0197 | 96.0000 | 88.20% |
|  | CCFM16gene0241 | 96.4072 | 84.32% |
|  | CCFM16gene1336 | 97.4052 | 81.08% |
|  | CCFM16gene1021 | 94.7581 | 84.89% |
| FBJ1M4 | FBJ1M4gene0006 | 96.9450 | 83.09% |
|  | FBJ1M4gene0335 | 95.4455 | 80.69% |
|  | FBJ1M4gene0645 | 96.3636 | 89.04% |
|  | FBJ1M4gene0695 | 98.4064 | 82.84% |
| FJSNT162 | FJSNT162gene0264 | 96.2810 | 86.69% |
|  | FJSNT162gene0630 | 97.2112 | 82.62% |
|  | FJSNT162gene0682 | 97.2727 | 90.10% |
|  | FJSNT162gene0853 | 97.7778 | 80.31% |
| FGZ612M3 | FJSNT162gene0298 | 96.6068 | 83.76% |
|  | FJSNT162gene0342 | 96.9035 | 88.93% |
|  | FJSNT162gene0499 | 95.7143 | 87.21% |
|  | FJSNT162gene0810 | 96.8379 | 80.12% |

**Table S3** Primer sequences used in the qPCR experiment

| Strain number | Gene number | Forward primer sequence（5’→3’） | Reverse primer sequence（3’→5’） |
| --- | --- | --- | --- |
| CCFM16 | *gene0197* | 5’-ACCCCGATCACCTGGAAAGGA-3’ | 3’-GTAGGAAACGACCGCAGGTAAG-5’ |
|  | *gene1021* | 5’-TCATCCATTCGGCCACCAAA-3’ | 3’-GTCGGCGGCGGACTGATCCA-5’ |
|  | *gene0241* | 5’-GACGATCAACGACGCGAACG-3’ | 3’-TCGCCGTAGGGCTGGATCTT-5’ |
|  | *gene1336* | 5’-ACATGGGATGTCAGCGACGA-3’ | 3’-CTGTCCTTGGGTGAACGGTTC-5’ |
| FBJ1M4 | *gene0006* | 5’-ACCCCGATCACCTGGAAAGGA-3’ | 3’-GTAGGAAACGACCGCAGGTAAG-5’ |
|  | *gene0335* | 5’-TCATCCATTCGGCCACCAAA-3’ | 3’-GTCGGCGGCGGACTGATCCA-5’ |
|  | *gene0645* | 5’-GACGATCAACGACGCGAACG-3’ | 3’-TCGCCGTAGGGCTGGATCTT-5’ |
|  | *gene0695* | 5’-ACATGGGATGTCAGCGACGA-3’ | 3’-CTGTCCTTGGGTGAACGGTTC-5’ |
| FJSNT162 | *gene0264* | 5’-ACCCCGATCACCTGGAAAGGA-3’ | 3’-GTAGGAAACGACCGCAGGTAAG-5’ |
|  | *gene0630* | 5’-TCATCCATTCGGCCACCAAA-3’ | 3’-GTCGGCGGCGGACTGATCCA-5’ |
|  | *gene0682* | 5’-GACGATCAACGACGCGAACG-3’ | 3’-TCGCCGTAGGGCTGGATCTT-5’ |
|  | *gene0853* | 5’-ACATGGGATGTCAGCGACGA-3’ | 3’-CTGTCCTTGGGTGAACGGTTC-5’ |
| FGZ612M3 | *gene0298* | 5’-ACCCCGATCACCTGGAAAGGA-3’ | 3’-GTAGGAAACGACCGCAGGTAAG-5’ |
|  | *gene0342* | 5’-TCATCCATTCGGCCACCAAA-3’ | 3’-GTCGGCGGCGGACTGATCCA-5’ |
|  | *gene0499* | 5’-GACGATCAACGACGCGAACG-3’ | 3’-TCGCCGTAGGGCTGGATCTT-5’ |
|  | *gene0810* | 5’-ACATGGGATGTCAGCGACGA-3’ | 3’-CTGTCCTTGGGTGAACGGTTC-5’ |
| Reference gene primers | | 5’-ACTCCTACGGGAGGCAGCAG-3’ | 3’-ATTACCGCGGCTGCTGG-5’ |

Thermocycling conditions: 95 °C for 30 s, followed by 30 cycles of 95 °C for 10 s and 60 °C for 10 s. For the melting curve, the temperature was raised from 65 °C to 95 °C in 0.5 °C increments every 5 s.


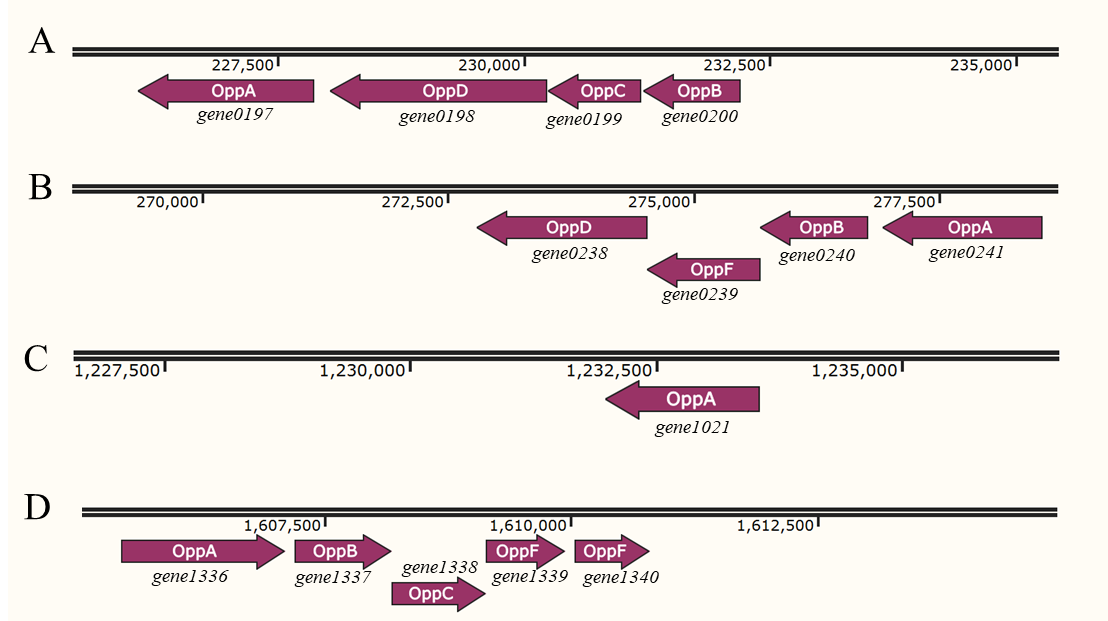


**Fig. S1** Opp system gene operons map of *B. bifidum* CCFM16. A) The operon containing OppA expressed by *CCFM16gene0197*; B) The operon containing OppA expressed by *CCFM16gene0241*; C) The operon containing OppA expressed by *CCFM16gene1021*; D) The operon containing OppA expressed by *CCFM16gene1336*

**Fig. S2** Relative expression level of *OppA* in *B bifidum* CCFM16, FBJ1M4, FJSNT162, FGZ612M3
